# Pasteur and Hydrophobia

**Published:** 1889-07

**Authors:** 


PASTEUR AND HYDROPHOBIA.—It is now asserted that as many as 136 deaths have followed Pasteur's innoculations for hydrophobia. Can't this *Madness* be stopped?

